# Building High‐Rate Nickel‐Rich Cathodes by Self‐Organization of Structurally Stable Macrovoid

**DOI:** 10.1002/advs.201902844

**Published:** 2020-02-11

**Authors:** Sujith Kalluri, Hyungyeon Cha, Junhyeok Kim, Hyomyung Lee, Haeseong Jang, Jaephil Cho

**Affiliations:** ^1^ Department of Energy Engineering School of Energy and Chemical Engineering Ulsan National Institute of Science and Technology (UNIST) 50 UNIST‐gil Ulsan 44919 Republic of Korea; ^2^ Department of Electronics and Communication Engineering School of Engineering and Applied Sciences SRM University‐AP Amaravati 522502 India

**Keywords:** high‐power lithium ion batteries, Kirkendall effect, LiNi_0.6_Co_0.2_Mn_0.2_O_2_, macrovoid structure, one‐pot synthesis

## Abstract

Nickel‐rich materials, as a front‐running cathode for lithium‐ion batteries suffer from inherent degradation issues such as inter/intragranular cracks and phase transition under the high‐current density condition. Although vigorous efforts have mitigated these current issues, the practical applications are not successfully achieved due to the material instability and complex synthesis process. Herein, a structurally stable, macrovoid‐containing, nickel‐rich material is developed using an affordable, scalable, and one‐pot coprecipitation method without using surfactants/etching agents/complex‐ion forming agents. The strategically developed macrovoid‐induced cathode via a self‐organization process exhibits excellent full‐cell rate capability, cycle life at discharge rate of 5 C, and structural stability even at the industrial electrode conditions, owing to the fast Li‐ion diffusion, the internal macrovoid acting as “buffer zones” for stress relief, and highly stable nanostructure around the void during cycling. This strategy for nickel‐rich cathodes can be viable for industries in the preparation of high‐performance lithium‐ion cells.

## Graphical abstract

A nickel‐rich cathode with a nanoparticle‐buffer layer induced macrovoid is developed via a one‐pot coprecipitation method without using surfactants/etching agents. The initial nanosized primary particles are dispersed toward the surface during the calcination process, which induces the nanoparticle‐based buffer zone and the structural stability. The prepared cathode material demonstrates a high specific capacity with strong structural integrity under the high C‐rate.

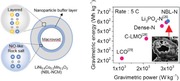

## 1 Introduction

The worldwide demands for fast‐charging portable devices and battery electric vehicles necessitated the development of high‐energy and high‐power lithium‐ion batteries (LIBs) as a power source. However, conventional nickel‐rich cathode materials have been limited in terms of sluggish intercalation kinetics caused by their intrinsic atomic structure and serious morphological/structural collapse under the high current operation. In these lines, there have been numerous attempts to improve the material stability by employing simple reheat treatment, surface coating/doping, concentration gradients, and controlling the morphology of the Ni‐rich particles.^1^, ^2^, ^3^, ^4^, ^5^, ^6^, ^7^, ^8^, ^9^, ^10^, ^11^, ^12^ However, though such strategies of the cathode particles significantly improved prolonged cycle life at the varied temperature conditions, there have been no remarkable improvements regarding the rate capability at high current operations, which is the point of interest for present electrical devices.

To overcome these issues, there have been two distinct approaches for the development of high‐power LIBs: electrode design and particle engineering. With respect to electrode design, previous study explored to shorten electron transport and lithium ions pathway by thinning the electrode, modifying current collectors, or adjusting the amount and morphology of conductive materials. At the same time, from the particle‐level perspective, fast charging and power capability have been improved by tuning the morphology and size of the cathode materials.^13^, ^14^ For example, the morphology engineering of cathode particle into hollow or porous structure and smaller particle size of <5 µm have been of great interest for high‐rate capability due to the effective utilization of active materials' surface area, well penetration of electrolyte, and thereby better Li‐ion mobility. However, realization of aforementioned approaches usually accompanies additional synthesis step and introduction of surfactants/etching agents/complex‐ion forming agents. Moreover, the point often overlooked is that these approaches could not ensure the structural stability under the harsh operation condition, demonstrating severe phase transition of the Ni‐rich cathode materials which lead to cell failure.

In this regard, nanostructure design should also be considered for the practical application of high‐power Ni‐rich cathode materials. Since preforming a rock‐salt and spinel phase at the surface reinforces the atomic structure by mitigating propagation of cation mixing layer, we emphasize that intentionally generated epitaxial phase could strongly stabilize the nanostructure of the Ni‐rich cathode materials. In short, morphology engineering with atomic structure design in a feasible way is a key parameter to guarantee the high‐power characteristic of the cathode material.

Here, we have developed complete hollow microsphere LiNi_0.6_Co_0.2_Mn_0.2_O_2_ with structurally stable nanoparticle buffer layer (NBL‐nickel‐based cathode (NCM)) surrounding the macrovoid in the core. The synthesis is self‐organization process based on delicate coprecipitation reaction without any mediating agent, followed by simple calcination procedure to develop macrovoid‐induced morphology of NBL‐NCM particle. During the calcination process, varied diffusion flux among the atoms results in oxygen deficient rock‐salt phase at the interface. This preformed rock‐salt phase around the macrovoid named as nanoparticle buffer layer provide structural rigidity of particles even after harsh electrochemical tests. Furthermore, this unique morphology of shell around the macrovoid facilitates rapid Li‐ion transport by increasing the active surface area and permeability.

## 2 Results and Discussion

**Figure 1:**
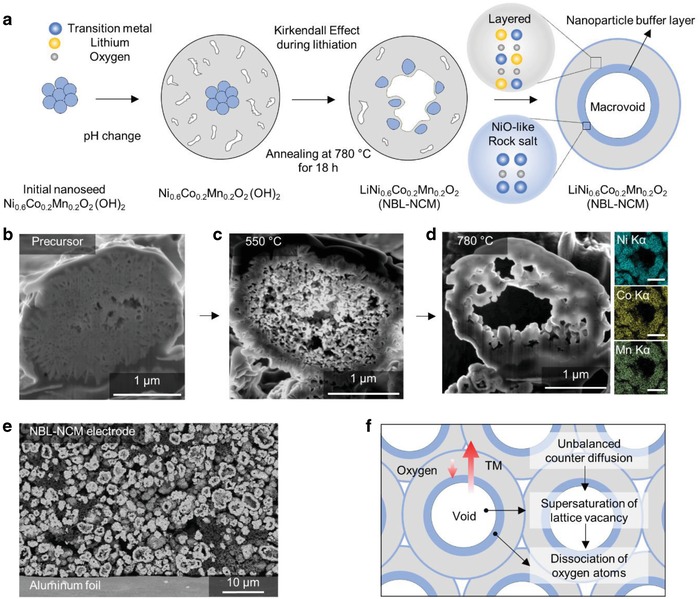
Schematic of NBL‐NCM synthesis and its significance. a) Schematic of the preparation of NBL‐NCM. FIB cross‐sectioned images of b) NBL‐NCM hydroxide precursor, c) lithiated NBL‐NCM while calcination at 550 °C, and d) completely lithiated NBL‐NCM at 780 °C depicting the macrovoid with nanoparticle buffer layer with EDX images of NBL‐NCM with uniform distribution of nickel and cobalt elements. e) Cross‐section image of NBL‐NCM electrode. f) Schematic of the void generation process.

Our strategy to prepare self‐organized hollow microsphere NBL‐NCM is illustrated in **Figure** **1**a. The Ni_0.6_CO_0.2_Mn_0.2_(OH)_2_ precursor was prepared with two steps of coprecipitation method in an equivalent batch reactor. The initial pH value for 20 min was set to 11.8 to synthesize the nanoseed precursor without feeding an ammonia solution, followed by change of pH to 10.8. The pH value and the amounts of ammonia solution affects to the morphological properties of the hydroxide precursor. Therefore, we intentionally adjusted the initial co‐precipitation step without ammonia solution for fabrication of nanosized and spherical seed to initiate the self‐organization during the calcination process. The self‐organization process in NBL‐NCM is induced by Kirkendall effect, which is facilitated in nanosized spherical particles. However, conventional NCM precursor based on coprecipitation method is demonstrated with densely packed microscale rod or plate‐like morphology since NH^3+^ ions fed in reactor act as a chelating agent, and Kirkendall effect hardly work in such environment. The careful tuning of these parameters resulted in the 4 µm sized spherical precursor particles with core and shell consist of fine nanoseed particles and nanoplate‐like structures, respectively. The initial nanoseed precursor in the core gradually diffuses toward the surface during the calcination process with lithium sources. Finally, the particle is transformed to completely hollow structure with macrosized void. The corresponding results are confirmed through focused ion beam (FIB) cross‐section image and energy dispersive X‐ray spectroscopy (EDX) of uniform distribution of Ni, Co, and Mn in Figure 1b–d, and the detailed void formation mechanisms are shown in Figure S1a–h of the Supporting Information. This uniform void structure was confirmed in the whole particles when it was fabricated to high‐energy electrode (Figure 1e). Surprisingly, the macrovoid formation is entirely self‐organization process without any mediating agent, analogous to the Kirkendall effect and Ostwald ripening phenomenon. The oppositely diffused transition metal atoms and oxygen atoms led to the supersaturation of lattice vacancy in an inner void because of the unbalanced counterdiffusion of them. Then, the dissociated oxygen atoms from the lattice diffuse through the gas‐solid interface, resulting in macrovoid surrounding oxygen deficient rock‐salt and disordered layered structure named as nanoparticle buffer layer (Figure 1f).

**Figure 2:**
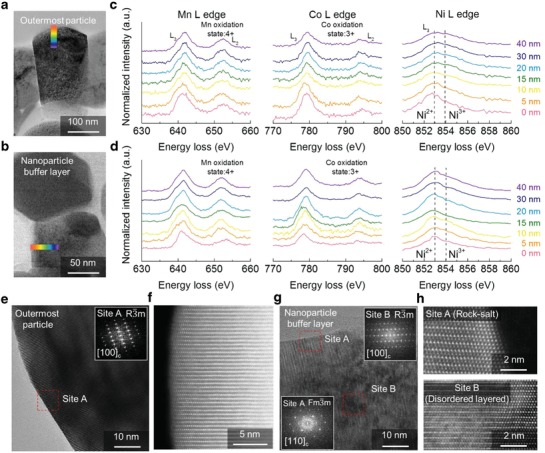
Atomic structure of NBL‐NCM. HR‐TEM images of a) outermost particle and b) nanoparticle buffer layer in NBL‐NCM and c,d) corresponding EELS profiles depicting the change in the oxidation states of Mn, Co, and Ni, respectively. f,h) HAADF‐STEM images of selected regions in (e) and (g) of outermost particle and nanoparticle buffer layer, respectively, showing their atomic level structures.

Such intrinsic properties of the NBL‐NCM were inspected by high‐resolution transmission electron microscopy (HR‐TEM) with electron energy loss spectroscopy (EELS). The EELS profiles of nickel, cobalt, and manganese show the variation of oxidation number; thus, we could correlate the oxidation number of transition metals in the surface and the primary particles near the void by the ratio of integrated intensities of L_3_ and L_2_ (**Figure** **2**a,b). Since we thoroughly controlled the nanostructure of the buffer layer and the outermost particles, the distribution of oxidation number according to the position in the secondary particle was intensively investigated. Noticeably, the valence state of nickel ions for the outermost particle was distinct from that of nanoparticle buffer layers, whereas no discernable change was observed in the cobalt and manganese ions. In terms of the oxidation state from the L_3_ peak at Ni–L edge in the outermost particle, the EELS spectra show the conventional behavior of NCM particle, where the divalent nickel ions are rich in the surface, followed by coexistence of divalent and trivalent nickel ions in the bulk structure (Figure 2c). The spontaneous reduction of trivalent nickel ions from the surface leads to the divalent nickel‐rich surface during the synthesis process.^15^, ^16^ On the contrary, the divalent nickel ions are dominant along the whole nanoparticle in the buffer layer near the macrovoid (Figure 2d). These results indicate the nanoparticle buffer layers near the macrovoid could be existed as NiO‐like phase. Since the self‐organization process induces oxygen‐deficient region in the nanoparticle buffer layer due to the varied diffusion flux of transition metal and oxygen atoms, the valence state of the nickel ions simultaneously changed to the divalent to build stable nanostructure. Accordingly, the high‐angle annular dark field scanning transmission electron microscope (HAADF‐STEM) images clearly show the nanostructure of the particles in the outermost and the buffer layers. In contrast to the well‐ordered layered structure in the outermost particles, relatively thick rock‐salt and disordered layered structure were observed in the buffer layer (Figure 2e–h). This inherent structure differences were originated from the morphology control during the initial primary particle formation step targeting self‐organization of the precursor. The nanoseed in the precursor core triggers diffusion of transition metal atoms and oxygen atoms oppositely during the calcination, resulting in supersaturation of lattice vacancy in the inner void.^17^, ^18^, ^19^, ^20^ Then, the dissociated oxygen atoms toward the core of the particle eventually lead to the oxygen deficient rock‐salt phase near the void. As is well known, the propagation of rock‐salt phase usually deteriorates the Li‐ion diffusivity by formation of an ionically insulated passivation layer, thus, highly thick rock‐salt phase at the surface increases the cathode overpotential upon the electrochemical cycling. However, several studies reported the preformed NiO‐like rock‐salt phase could enhance the structural stability under the long‐term cycling rather acting as a resistance layer.^9^, ^21^, ^22^, ^23^, ^24^, ^25^ In this regard, NBL‐NCM is strategically designed with double surface system, whereby the inside the shell is composed of preformed NiO‐like phase and well‐ordered layered structure at the outermost region.

To verify influence of chemical and structural properties of the NBL‐NCM on electrochemical performance, NBL‐NCM were electrochemically characterized in the 2032R coin‐type half‐cell and pouch‐type full‐cell, where the both cathodes loading level and density was 11.0 ± 0.2 mg cm^−2^ and ≈3.0 g cm^−3^, respectively. The prepared NBL‐NCM electrodes were subjected to the ion‐milling, and the cross‐section morphology was analyzed before and after electrode pressing process. Figure S2 of the Supporting Information shows the uniform distribution of macrovoids in the secondary particles, and these voids were not fractured, maintaining the hollow structure even after pressing to high loading density. For direct comparison, densely packed LiNi_0.6_Co_0.2_Mn_0.2_O_2_ (NCM) was synthesized by traditional coprecipitation method. Both NCM and NBL‐NCM samples show spherical morphology with the secondary particle size of ≈4 µm (Figure S3a–f, Supporting Information). The phase and lattice parameters were estimated by the X‐ray diffraction pattern and least‐square method, respectively (Figure S4, Supporting Information). Both samples were indexed to the hexagonal layered structure of *R* $\bar{3}$ *m* space group, consistent with the general patterns of NCM cathode materials.^3^, ^26^, ^27^ Furthermore, the well‐ordered atomic structure for NCM was confirmed by the HR‐TEM image (Figure S5, Supporting Information). The detailed physical properties are described in Table S1 of the Supporting Information.

**Figure 3:**
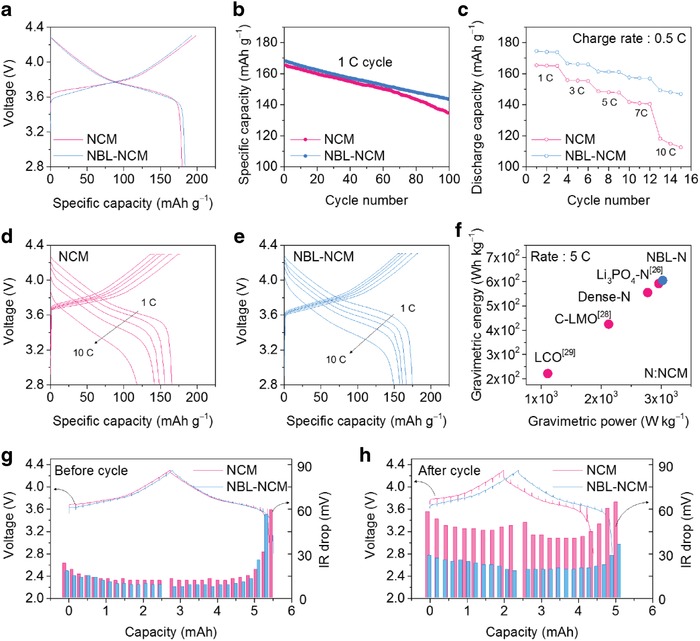
Electrochemical characterization of NCM and NBL‐NCM. a) Formation cycle and b) long‐term cycle of NCM and NBL‐NCM in the voltage range of 2.8–4.3 V versus Li/Li^+^. c) The rate capability of NCM and NBL‐NCM half cells at the constant charge rate of 0.5 C and the respective discharge rate at varied C‐rates from 1 to 10 C in the voltage range of 2.8–4.3 V versus Li/Li^+^ and d,e) their voltage profiles at various C‐rates showing the overpotentials. f) Ragone plot illustrating the outstanding performance of NBL‐NCM in terms of gravimetric energy and power densities at high C‐rate (5 C) when compared to other class of NCM and different cathode materials in the literature. The GITT results of NCM and NBL‐NCM g) before cycle and h) after cycle. Abbreviations: LMO – LiMn_2_O_4_, LCO – LiCoO_2_.

The initial discharge capacity of NCM and NBL‐NCM was 179 and 183 mAh g^−1^, respectively, with the initial Coulombic efficiencies of 90% and 95% (**Figure** **3**a). The significant improvements in the capacity value and the Coulombic efficiency are associated with the better electrolyte percolation through the internal void and shortened Li‐ion diffusion pathways in the highly activated surface area. Accordingly, the Brunauer–Emmett–Teller (BET)‐specific surface area of NBL‐NCM was estimated to 2.02 m^2^ g^−1^, which is four times higher value than that of conventional NCM particle (Table S1, Supporting Information). Also, it is noted that the partially formed rock‐salt structure in NBL did not act as resistance layer, since NBL was epitaxially formed above layered structure during the synthesis process at high temperature. Under this circumstance, the nanostructure was highly stabilized, and further increased the nanostructure stability upon the current flow. Also, these properties lead to long‐term cycle stability of NBL‐NCM (Figure 3b). In particular, the rate capability of the cathode material is crucial for the realization of the high‐power lithium‐ion batteries. So, we severely demonstrated the rate performance of the cathodes under the various current rate conditions and the Li‐ion diffusivity characteristics. Interestingly, NBL‐NCM showed enhanced rate capability and capacity values at all C‐rates when compared to NCM (Figure 3c). For instance, the discharge capacities of NBL‐NCM at 1 and 10 C are 175 and 151 mAh g^−1^, respectively, are much higher than that of NCM (164 and 112 mAh g^−1^) with low overpotentials for each cyle (Figure 3d,e). Furthermore, the charge rate capability was also remarkable in the NBL‐NCM (Figure S6a–d, Supporting Information). More importantly, we compared the gravimetric energy and power density of the NBL‐NCM at a high C‐rate with those of Ni‐rich and other cathode materials (LiMn_2_O_4_, LiCoO_2_, etc.) represented as a Ragone plot to demonstrate high‐power capability among the cathode materials (Figure 3f).^26^, ^28^, ^29^ The respective average discharge voltage and maximum obtainable electrode density values were considered for all cathode materials while calculation of their specific energy and power values are referred in Equation (S1) of the Supporting Information. Interestingly, the NBL‐NCM outperformed than the other particle morphologies or the surface‐coated showing the specific energy and power of ≈600 Wh kg^−1^ and ≈3 kW kg^−1^, respectively, at 5 C rate. Such superior electrochemical performance was attributed to the nanoparticle buffer layer surrounding well‐ordered layered structure with the unique morphology of NBL‐NCM which could effectively relieve the stress under the high current flow.

Moreover, we confirmed superior electrochemical characteristics of the NBL‐NCM was preserved after the rate test through galvanostatic intermittent titration technique (GITT) results Remarkably, NBL‐NCM showed significantly reduced overall overpotential and IR drop when compared to that of NCM. These reduced overpotentials suggest the preformed rock‐salt structured buffer layer near the void reinforced the structure stability rather than working as resistance layer during high C‐rate cycling.

**Figure 4:**
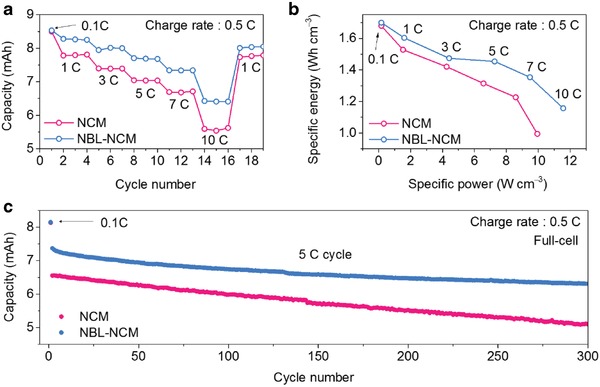
Full‐cell evaluation of NCM and NBL‐NCM. a) The rate capability of NCM and NBL‐NCM pouch‐type full‐cells at the constant charge rate of 0.5 C and the respective discharge rate at varied C‐rates from 1 to 10 C in the voltage range of 2.8–4.2 V. b) Volumetric energy and power values at varied C‐rates of NCM and NBL‐NCM full‐cells. c) Long‐term cycle performance of NCM and NBL‐NCM full‐cell at discharge rate of 5 C and constant charge rate of 0.5 C.

To provide further insight into the commercial viability, the pouch type full‐cells (Figure S7a, Supporting Information) were assembled with the NBL‐NCM and NCM as cathode materials and natural graphite as an anode material. The full‐cell design specifications are mentioned in Table S2 of the Supporting Information. Initially, the 8.5 mAh full‐cells were activated with the formation cycle at 0.1 C including the degassing step (Figure S7b, Supporting Information). The rate capability tests were performed in the varied C‐rates from 0.1 to 10 C and as shown in **Figure** **4**a. The NBL‐NCM cells showed an enhanced overall rate performance and excellent capacity retention even after 10 C rate. When it is converted to the volumetric energy and power densities, the NBL‐NCM full‐cells exhibited 1.16 Wh cm^−3^ and 11.6 W cm^−3^ at 10 C, respectively, which is comparable value to that of NCM (0.99 Wh cm^−3^ and 9.9 W cm^−3^) as represented in Figure 4b owing to its lower full‐cell capacity under the same environment. The long‐term rate and cycle performance of the NBL‐NCM were further validated at charge rate 0.5 C and discharge rate 5 C in the voltage range of 2.8–4.2 V. Notably, there is an appreciable improvement in cycle performance of NBL‐NCM in comparison with NCM over 300 cycles (Figure 4c). The difference in the initial discharge capacity of both cells during 5 C cycle implies that the NCM could not accommodate the fast Li‐ion mobility at high rates, whereas, NBL‐NCM was proved to facilitate fast Li‐ion transport due to its high surface area and better surface permeability. In terms of the better cyclability under the high‐current operating conditions, the presence of NBL was highly regarded by mitigating morphological and nanostructural distortion. Hence, these observations were mainly originated from the synergy effect of high surface area of unique morphology with structurally stable buffer zone surrounding the macrovoid of the particle, which can provide better percolation of electrolyte with much faster Li‐ion mobility and proven high structural integrity at high‐current operating conditions. In addition, the NBL‐NCM showed stable electrochemical performance under the high temperature of 45 °C (Figure S8, Supporting Information).

**Figure 5:**
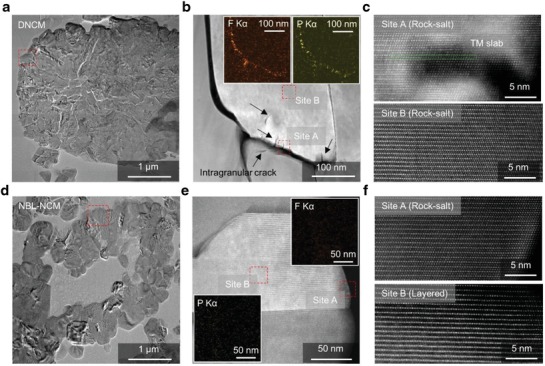
Postmortem analysis of the NCM and NBL‐NCM electrodes after high rate cycle test. a) The low‐magnified HAADF‐STEM image of the NCM secondary particle. b) The magnified image of NCM showing the intragranular, intergranular, and surface microcracks formation (insets: EDXS images of the F and P mapping). c) The STEM image representing the rock‐salt crystalline structure of selected region site A and site B of (b). d) The low‐magnified HAADF‐STEM image of the NBL‐NCM secondary particle proved the retainment of macrovoid structure and structure stability after high rate cycling. e) The high‐resolution image of the marked region in (d) (insets: EDX images of the F and P mapping). f) The STEM images showing the crystalline structure at site A (surface) and site B (core) of NBL‐NCM primary particle as rock‐salt and layered structures, respectively.

Obviously, outstanding morphological and structural properties of the NBL‐NCM were guaranteed even after harsh electrochemical tests. Following the high‐rate cycling test, the pouch‐type full‐cells were disassembled to investigate the morphology of secondary particles, chemical stability, and the integrity among the primary particles. The HAADF‐STEM analysis was employed to understand the crystalline structure variations of the NCM and NBL‐NCM samples after high rate cycling. The high magnified HR‐TEM image (**Figure** **5**b) of selected region in Figure 5a depicts the history of the various microcrack formations at the intragranular, intergranular, and surface levels of the NCM primary particles. This explains the severity of the electromechanical stress induced on the particles during high rate operations and led to the deterioration of the crystalline structure to the rock salt phase and, thereby cracking the primary particle and blocking of the Li‐ion pathways (Figure 5c).^16^, ^30^, ^31^ Conversely, NBL‐NCM reported excellent structural stability with the perfect hollow‐structure and integrity among the primary particles (Figure 5d). There is no evidence of the microcracks at the primary and secondary particle levels, suggesting the influence of internal macrovoid in the hollow‐structure tends to withhold the electromechanical stress while the rigid nanostructure of the buffer layer at the NBL‐NCM maintains the structural stability (minimize the microcrack initiation) and better Li‐ion mobility over high rate cycling. As a result, only slight NiO‐like rock‐salt phase on the surface was propagated, thus the inner core of the particle maintained the excellent layered structure as shown in Figure 5e,f even after high rate operations under the high cathode loading level and density.

Furthermore, as shown in cross‐section image in Figure S9a,b of the Supporting Information and the inset of Figure 5b,e, the secondary particles of NCM suffered from the severe intergranular cracks and lead to the additional electrolyte decomposition between the cracks. The cracks in the cathode particle could trigger the continuous growth of the solid‐electrolyte interphase (SEI) layer with the thickness of 30 nm as evidenced from the EDX mapping images corresponding to phosphorous and fluorine. These cycled samples are subjected to the Ar etching and then X‐ray photoelectron spectroscopy (XPS) peak profiles (Figure S9c,d, Supporting Information) of the fluorine (F) and phosphorous (P) were obtained for both samples and no variation in the chemical composition (i.e., LiF, Li*_x_*PF*_y_*, and Li*_x_*PO*_y_*F*_z_*) was found despite the morphology change. Such SEI layer across the primary particles could significantly cause sluggish Li‐ion diffusion, and thus diminish the high‐rate performance of NCM. In terms of NBL‐NCM, since the active surface area was greatly increased by hollow morphology, there has been also an increase of the sites for oxidative decomposition of electrolyte. Therefore, the SEI formation is critical for both samples in a different way after electrochemical cycling.

## 3 Conclusion

We have successfully designed the complete void structure with structurally stable buffer layer via the self‐organization process by using industrial‐scale, one‐pot, and uniquely modified coprecipitation method. The formation of void initiated by the Kirkendall porosity during the optimized calcination conditions, purposefully induced the reduced nickel oxidation state to preform thin cation‐mixing buffer layer near the void. The strategic design of nickel‐rich cathode set out superior electrochemical performance in terms of specific discharge capacity of 183 mAh g^−1^ with 95% Coulombic efficiency, volumetric energy and power density, and cyclic stability even at high C‐rate of 10 C under the high cathode loading level and density. These electrochemical enhancements are attributed to the preformed rock‐salt structured buffer layer that could stabilize the layered structure in the shell and facilitate fast Li‐ion transport at high rates. Notably, the present unique and facile synthesis technique without any reagents can significantly reduce the processing time and capital cost. Therefore, the commercially viable, scalable, and the high‐performing NBL‐NCM is believed to be applied in the high‐power lithium‐ion batteries for electronic/automotive applications.

## 4 Experimental Section

### NBL‐NCM and NCM Material Synthesis

The traditional coprecipitation method was employed to prepare NBL‐NCM cathode materials by using the transition metal (TM) solution precursor solution of nickel (II) sulfate hexahydrate (NiSO_4_·6H_2_O), cobalt (II) sulfate heptahydrate (CoSO_4_·7H_2_O), and manganese (II) sulfate (MnSO_4_·H_2_O) with concentration of 2 m a molar ratio of 0.6:0.2:0.2 and 2.5 m sodium hydroxide (NaOH) solution in the absence of ammonia solution (NH_4_OH). The solutions of TM and NaOH were fed into the continuous stirred tank reactor of 4 L capacity with the same feeding rate of 300 mL h^−1^ under the reaction temperature of 60 °C and the gradual variation of pH value from 12.0 to 10.6 to influence the morphology of primary particles. Finally, the obtained Ni_0.6_Co_0.2_Mn_0.2_(OH)_2_ precursor was washed several times with distill water and dried in oven at 80 °C overnight. The stoichiometric amounts of NBL‐NCM precursor and LiOH·H_2_O were mixed with a molar ratio of 1:1.05 and annealed at 780 °C for 18 h in oxygen atmosphere. For comparison, NCM precursor was prepared by same coprecipitation method with NH_4_OH solution and without varying the pH value during the coprecipitation experiment. Further lithiation conditions were same to that of NBL‐NCM.

### Material Characterization

The physical and structural properties of the prepared NCM and NBL‐NCM were tested with various characterization tools. The presence and quantity of residual free lithium compounds (LiOH, Li_2_CO_3_) were estimated by employing a potentiometric titrator (888 Titrando, Metrohm). For the measurement, the prepared materials were dispersed in the deionized water and the resultant solution was filtered. Rigaku D/MAX 2500 V/PC X‐ray diffractometer with Cu Kα radiation was used to obtain the X‐ray diffraction patterns of the powdered samples. The particle size distribution of the powdered samples was measured by using the Microtrac S3500 particle size analyzer. Inductively coupled plasma optical emission spectrometry (IRIS Intrepid II, USA) was used to estimate the elemental composition of the samples Pellet density measurements were carried out on 10 g active material 360 MPa pressure. Morphology and electrode thickness/cross‐section were observed by using scanning electron microscopy (Verios 460, FEI). FIB (Helios Nano Lab450, FEI) was used to realize the cross‐sectional morphology and the hollow‐structure of the cathode secondary particles. For TEM, the cross‐sectioned samples were thinned by using argon‐ion milling system (Model 1040 Nanomill, Fischione). The structural analysis including HAADF‐STEM was conducted by using a probe‐side aberration corrected TEM (JEM‐2100F, JEOL), whereas, an image‐side aberration corrected TEM (Titan G2 60–300, FEI) was employed to carry out the STEM‐EELS analysis. The dodeca‐pole aberration corrected TEM (JEM‐ARM300F, JEOL) was used to perform the EDX analysis at electron beam of 80 kV to prevent sample damage. BET surface area, pore size distribution, and porosity were measured using mercury porosimeter (Autopore V 9605, Micromeritics). XPS (Kratos Axis Ultra spectrometer with Al Kα radiation, *hν* = 1486.71 eV) measurements were carried out to explore surface of both samples after cycling. Macromode (≈4 mm × 4 mm) with Ar‐ion etching (with etching rate 0.5 nm min^−1^ for a silica patch) was conducted to support XPS in measuring the concentrations of transition metal elements at different depths from the surface into the bulk of the samples after high rate cycle test. Thermal properties of the delithiated electrodes (from disassembled cells fully charged to 4.3 V vs Li) were measured by differential scanning calorimetry (DSC1 star system, METTLER).

### Electrochemical Characterization for Half‐Cell Coin‐Cell Type

The electrodes for both samples were prepared from a slurry composition of active material: carbon black (Super‐P): polyvinylidene fluoride (PVDF) in the ratio 94:3:3 (wt%). First, PVDF was dissolved in *N*‐methyl pyrrolidinone and stirred for 5 min, and second, active material and Super‐P were mixed with the resultant solution and stirred at 7000 rpm for 10 min using homogenizer. The obtained slurry was coated on aluminum foil using doctor blading and dried at 110 °C for 2 h. After pressing, the circular electrode disks specifications are maintained as 14 mm in diameter with active material loading mass of ≈11 mg cm^−2^ and the electrode density was 3.0 g cm^−3^. Standard coin‐type half‐cells (2032R) were assembled in a glove box equipped with Argon gas flow, using lithium metal foil circular disks as a counterelectrode and 1.15 m LiPF_6_ in ethylene carbonate/ethyl methyl carbonate/dimethyl carbonate (3/4/3 vol%) (Panax Starlyte) as an electrolyte. Aging period of assembled coin‐cells was 24 h and WonATech (Korea) battery testers were used for all electrochemical tests. The galvanostatic charge–discharge behavior was carried out in the potential window of 4.3–2.8 V versus Li/Li^+^. The GITT was carried out at a constant current pulse of the 0.1 C rate for 10 min then rest for 55 min to stabilize the cell voltage between 2.8 and 4.3 V.

### Electrochemical Characterization for Full‐Cell Pouch Type

In the full‐cell configuration, the anode and cathode are natural graphite (BTR, China) and prepared NCM samples, respectively, and the electrolyte is same as that of half‐cell. The cathode electrode composition remained same as that of half‐cell type and the anode electrodes were prepared in the composition of 97 wt% natural graphite as active material, 1.5 wt% styrene‐butadiene rubber and 1.5 wt% carboxymethyl cellulose as binders. The pouch‐type full‐cells with N/P ratio 1.14, cathode and anode loading mass as 11.0 and 5.4 mg cm^−2^, respectively, were assembled in the dry room. Both cycle (at 25 and 45 °C) and rate tests (at 25 °C) were performed in the voltage range of 2.8–4.2 V using WonATech battery testers using the same method as that of half‐cells with the charging of pouch‐cells under CC–CV mode (constant current with each C rate and constant voltage at 4.2 V with a cut‐off current of 0.05 C).

## Conflict of Interest

The authors declare no conflict of interest.

## Supporting information

Supporting Information
