# Supplementary material for: Building High‐Rate Nickel‐Rich Cathodes by Self‐Organization of Structurally Stable Macrovoid
Source: Adv Sci (Weinh). 2020 Feb 11;7(7):1902844. doi: 10.1002/advs.201902844 (PMC7140999; doi:10.1002/advs.201902844)
Supplement: Supplementary file 1 — Supporting Information [file ADVS-7-1902844-s001.pdf]

Copyright WILEY-VCH Verlag GmbH & Co. KGaA, 69469 Weinheim, Germany, 2020.

ADVANCED SCIENCE
Open Access

Supporting Information

for *Adv. Sci.,* DOI: 10.1002/advs.201902844

Building High-Rate Nickel-Rich Cathodes by Self-Organization of Structurally Stable Macrovoid

*Sujith Kalluri, Hyungyeon Cha, Junhyeok Kim, Hyomyung Lee, Haeseong Jang, and Jaephil Cho**

((Supporting Information can be included here using this template))

Copyright WILEY-VCH Verlag GmbH & Co. KGaA, 69469 Weinheim, Germany, 2020.

Supporting Information

Building High-Rate Nickel-Rich Cathodes by self-organization of structurally stable macrovoid

*Sujith Kalluri, Hyungyeon Cha, Junhyeok Kim, Hyomyung Lee, Haeseong Jang, Jaephil Cho**

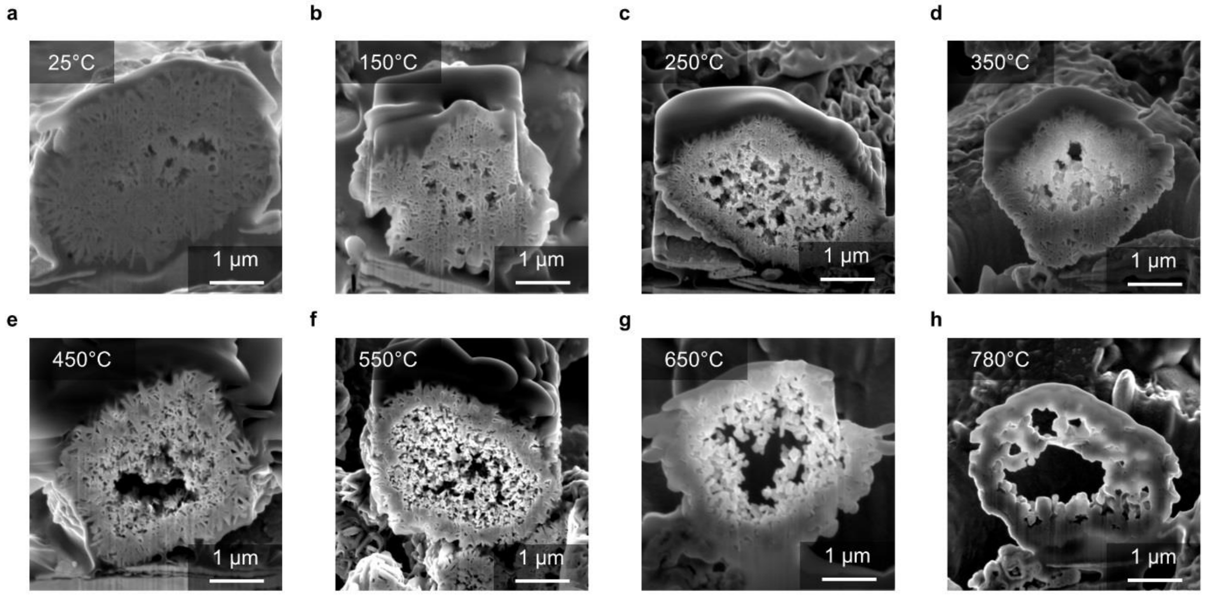

Figure S1. (**a**)-(**h**) Macro-void structure evolution by the FIB cross-sectioned images of NBL-NCM particles at the various stages of lithiation calcination process (from 25℃ to 780℃).

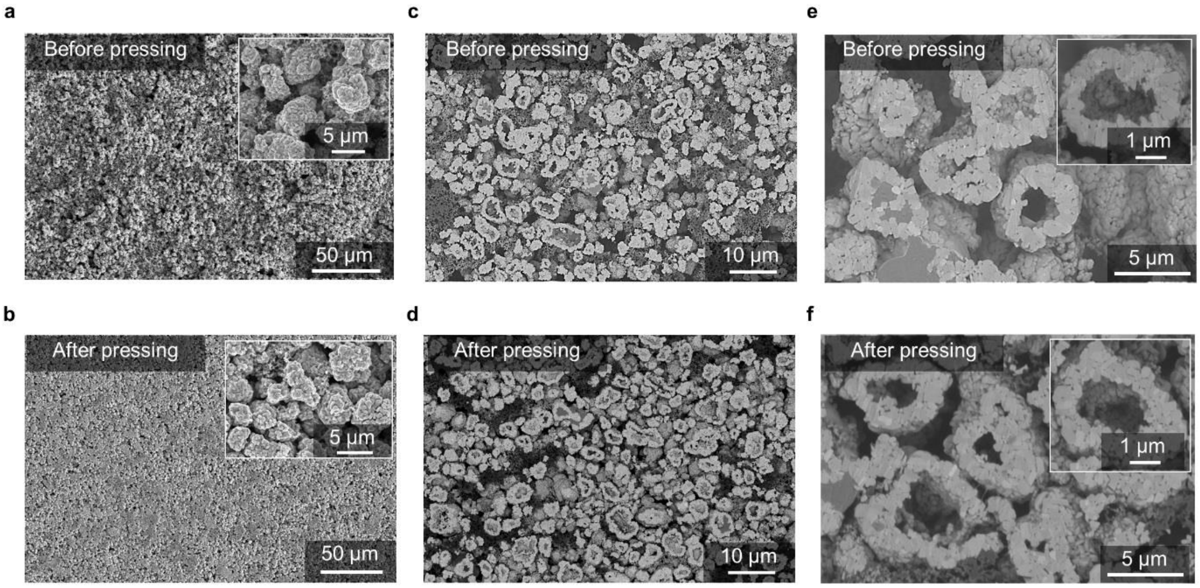

Figure S2. Structural properties of electrodes at electrode loading density of 3.0 g cm^{-3}. Top-view SEM images of the NBL-NCM electrodes (**a**) before and (**b**) after pressing, respectively with the highly magnified images in the inset. (**c**), (**e**) The ion-milled cross-sectioned SEM image of the electrode before pressing. (**d**), (**f**) The ion-milled cross-sectioned SEM image of the electrode after pressing depicting the structurally stable hollow-structured morphology.

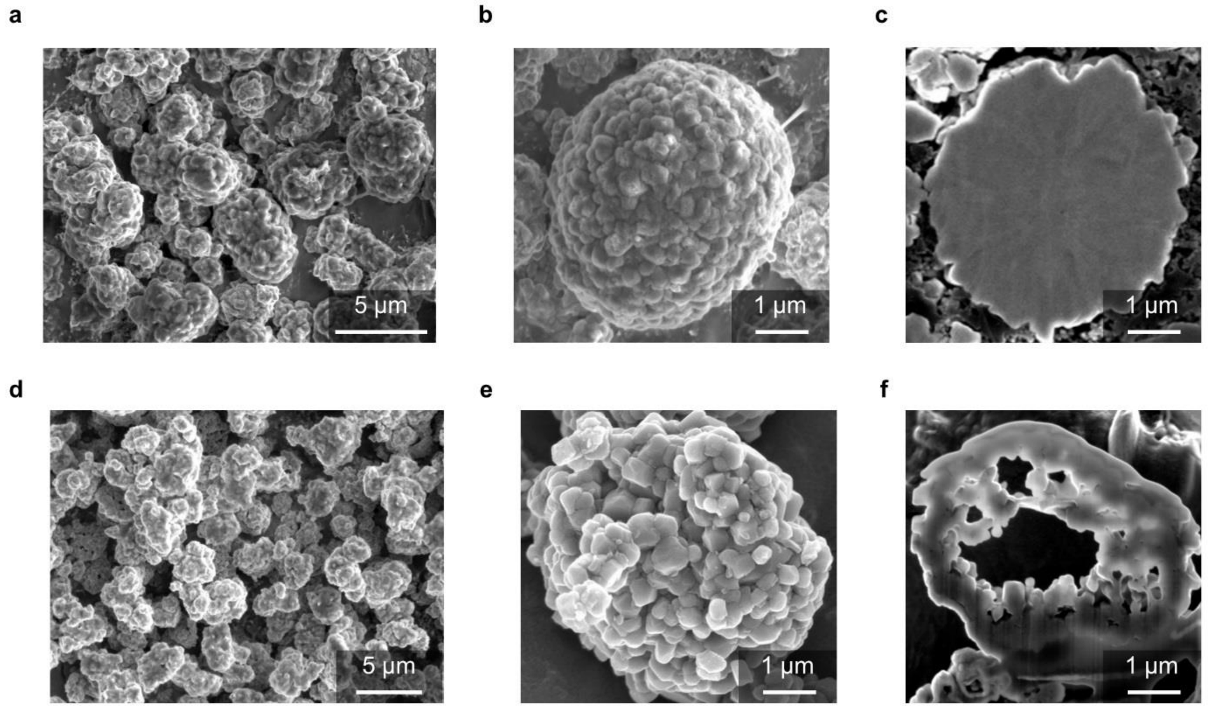

Figure S3. (**a**) SEM images of DNCM. (**b**) High-resolution SEM images of DNCM. (**c**) Cross-sectioned FIB images of DNCM. (**d**) SEM images of and NBL-NCM. (**e**) High-resolution SEM images of NBL-NCM. (**f**) Cross-sectioned FIB images of NBL-NCM.

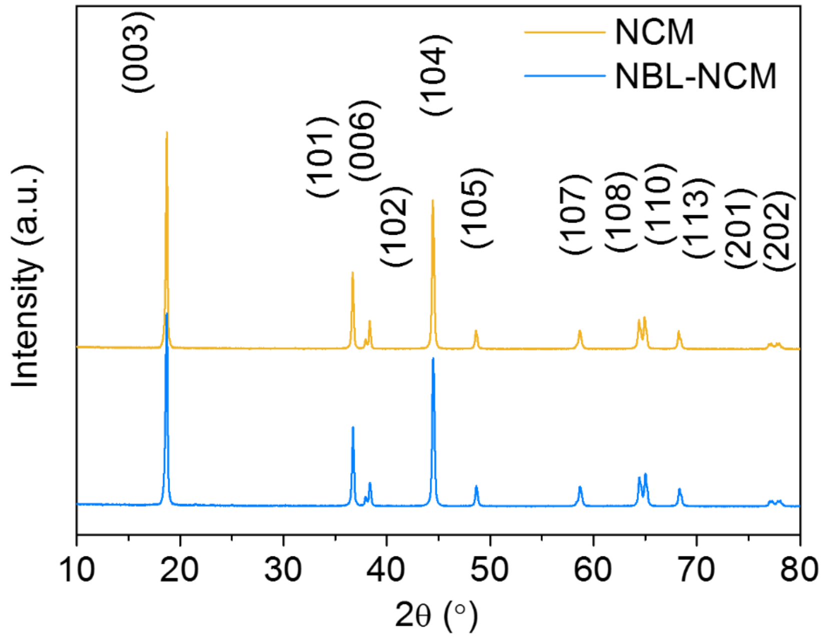

Figure S4. XRD patterns of DNCM and NBL-NCM powders.

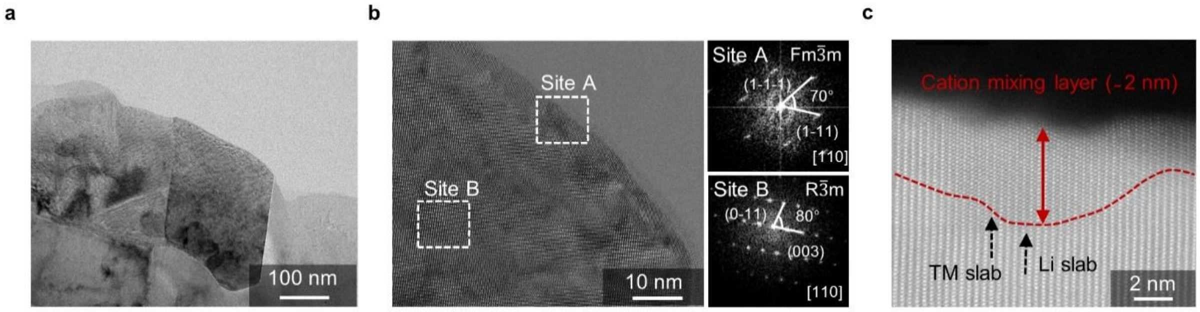

Figure S5. (**a**) The HAADF-STEM image of the DNCM particle. (**b**) The magnified image with FFT patterns of the selected regions site A (surface) and site B (core) of the DNCM as Fm3̄m phase rock-salt and R3̄m phase layered structure, respectively. (**c**) The STEM image of site A showing the cation-mixing layer on the surface of the DNCM particle.

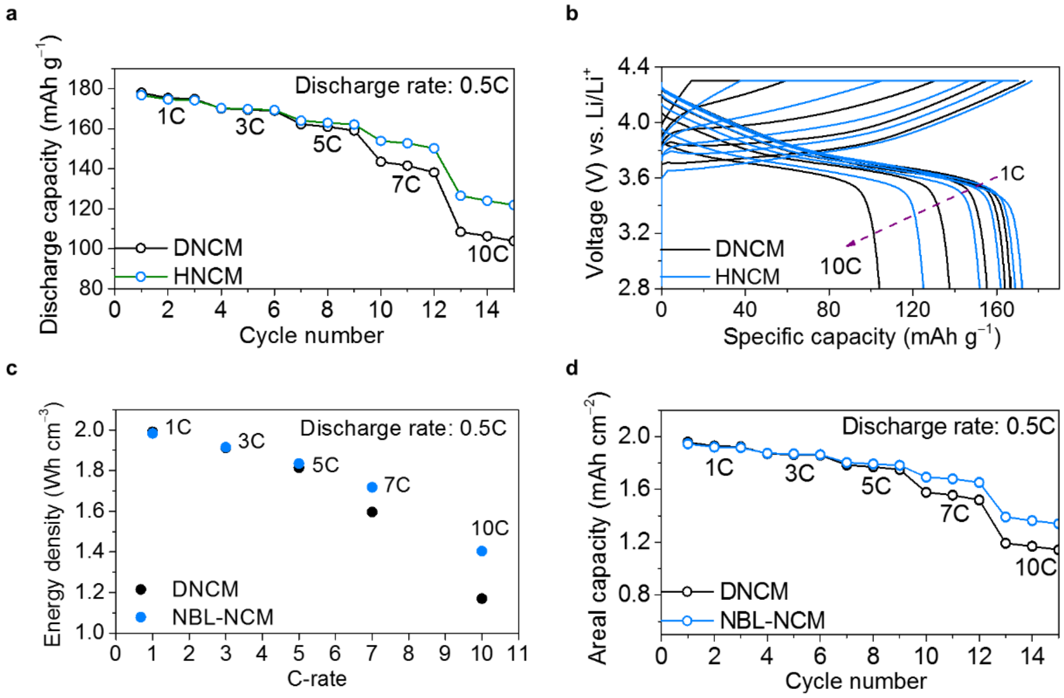

Figure S6. Electrochemical properties of DNCM and NBL-NCM half-cells at constant discharged rate 0.5 C. (**a**)-(**d**) Discharge capacity, voltage profiles, energy density, and areal capacity trends of half-cells charged at varies C-rates from 1 C to 10 C.

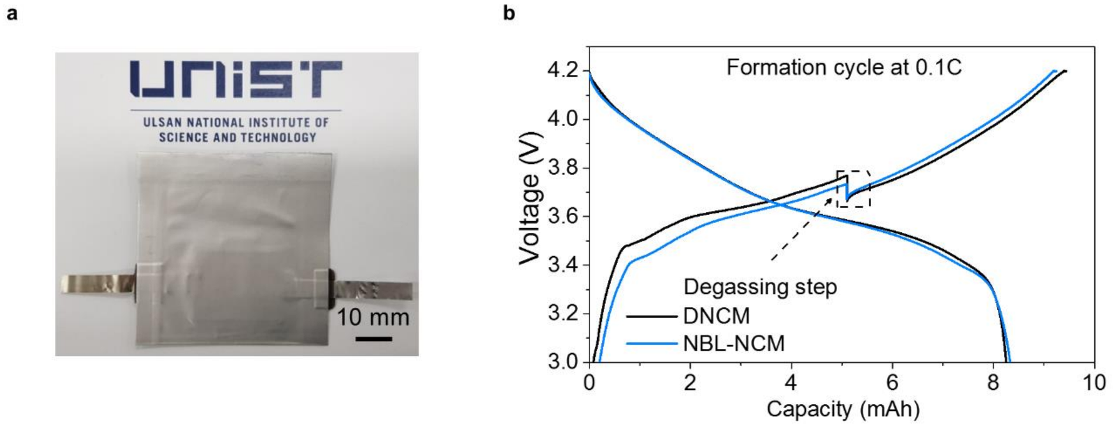

Figure S7. Electrochemical properties of DNCM and NBL-NCM pouch-type full-cells in the voltage range of 2.8 - 4.2 V *vs.* NG. (**a**) Photograph of Al pouch-type full-cell. (**b**) The full-cell formation cycles of DNCM and NBL-NCM at 25℃.

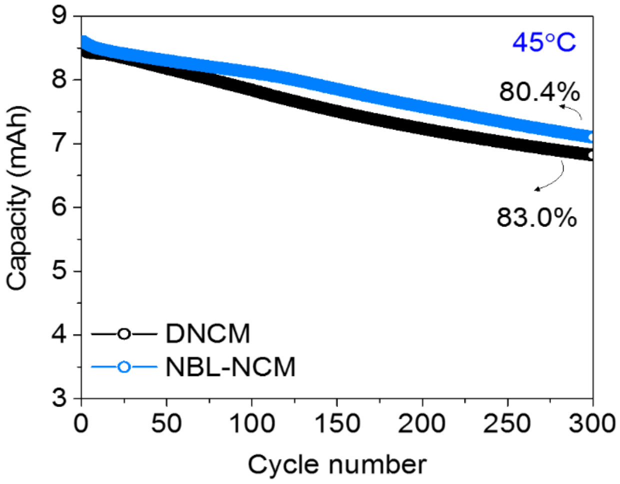

Figure S8. Cycle performance of DNCM and NBL-NCM full-cells at high temperature (45℃) and high rate (5 C discharge) for 300 cycles.

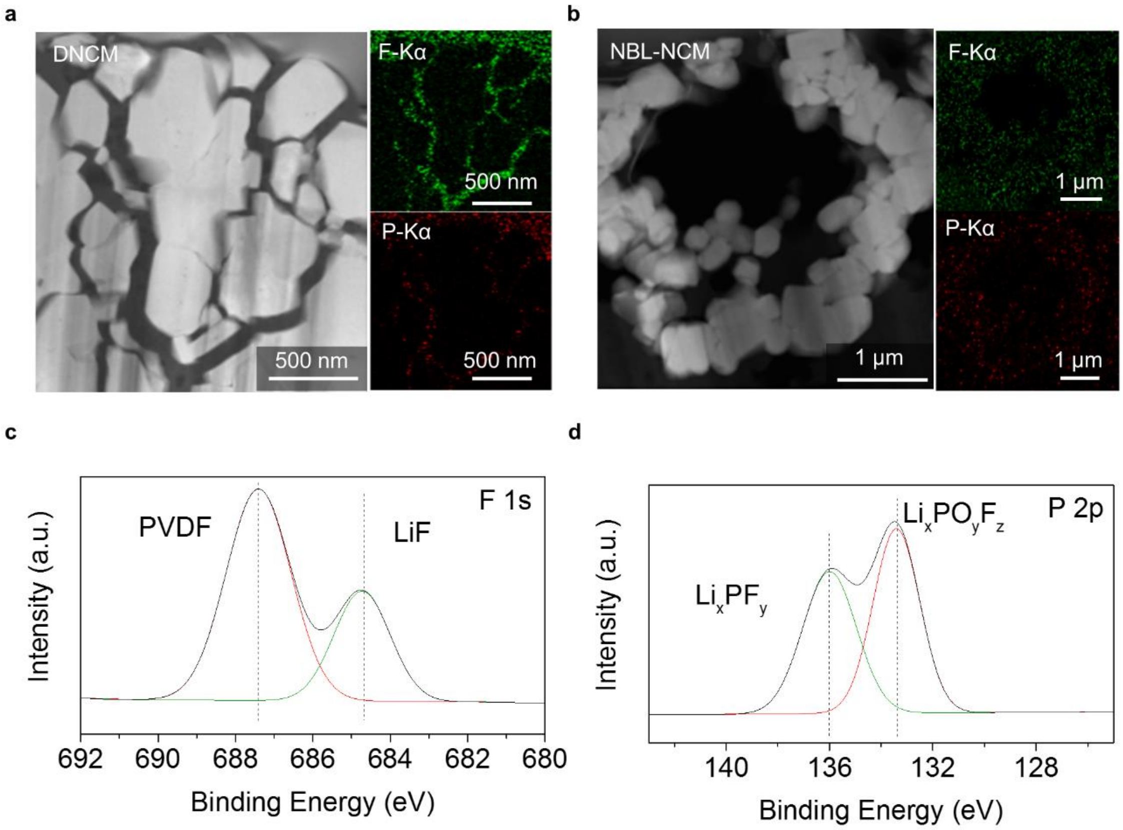

Figure S9. EDXS analysis after high rate cycle test. The HAADF-STEM image of the NCM622 secondary particle with the corresponding elemental mapping images. (**a**) DNCM. (**b**) NBL-NCM. (**c**), (**d**) XPS peak profiles of F and P in DNCM.

Table S1. Physical properties of DNCM and NBL-NCM powders.

Physical property		NCM	NBL-NCM
Residual Li (wt.%)		0.189	0.185
BET (m^2 g^{-1})		0.48	2.02
Tap density (g cm^{-3})		2.5	2.4
Particle size distribution	D_{10} (μm)	2.78	1.13
	D_{50} (μm)	4.83	4.28
	D_{90} (μm)	8.07	7.26
ICP	Li (ppm)	1.11	1.06
	Ni (ppm)	0.59	0.59
	Co (ppm)	0.20	0.20
	Mn (ppm)	0.20	0.20

Table S2. Design specifications of the pouch-type full-cell.

Specification	Value
Cathode loading level (mg cm^{-2})	11.0
Anode loading level (mg cm^{-2})	5.4
Cathode electrode density (g cm^{-3})	3.0
Anode electrode density (g cm^{-3})	1.5
Full-cell N/P ratio	1.14

Equation S1. The calculation of gravimetric and volumetric energy/power densities with respect to the cathode electrode

$$\text{Gravimetric energy density (Wh kg}^{-1}\text{)} = \frac{\text{(Cell capacity, mAh)} \times \text{(Average discharge voltage, V)}}{\text{(Cathode area, cm}^{2}\text{)} \times \text{(Loading level of cathode, mg cm}^{2}\text{)}}$$

$$\text{Volumetric energy density (Wh cm}^{-3}\text{)} = \frac{\text{(Cell capacity, mAh)} \times \text{(Average discharge voltage, V)}}{\text{(Cathode area, cm}^{2}\text{)} \times \text{(Thickness of cathode, μm)}}$$
